# Effects of Thermal Activation on Mechanical Performance and Sustainability of Slag-Based Geopolymers

**DOI:** 10.3390/ma18184419

**Published:** 2025-09-22

**Authors:** Lais Alves, Nordine Leklou, Fábio de Simone e Souza, Silvio de Barros

**Affiliations:** 1 Federal Center for Technological Education Celso Suckow da Fonseca (CEFET/RJ), Rio De Janeiro 20271-110, Brazil; fabio.souza@cefet-rj.br (F.d.S.e.S.); silvio.debarros@gmail.com (S.d.B.); 2 University of Nantes, École Centrale Nantes, CNRS, Institut de Recherche en Génie Civil et Mécanique, UMR 6183, 44600 Saint-Nazaire, France; nordine.leklou@univ-nantes.fr; 3 CESI LINEACT, 44600 Saint-Nazaire, France

**Keywords:** geopolymer, ground granulated blast furnace slag (GBFS), low-carbon construction materials, mechanical properties, porosity, shrinkage, sustainable binder, thermal curing

## Abstract

Ground granulated blast furnace slag (GBFS)-based geopolymers represent a viable binder system that combines mechanical efficiency with a significantly lower carbon footprint when compared to conventional Portland cement. This work examines how thermal curing between 20 °C and 80 °C affects setting time, mechanical performance, shrinkage, and porosity of GBFS-based geopolymers. Curing at 40 °C accelerated gel formation, yielding compressive strengths up to 71.9 MPa. This regime also reduced shrinkage and porosity. In contrast, curing at ≥60 °C caused structural degradation and reduced long-term performance. Statistical analysis (ANOVA and Tukey post hoc) confirmed significant effects of curing regime and age on performance. These findings provide key insights for optimizing thermal curing of slag-based geopolymers, supporting their deployment in environmentally responsible construction practices.

## 1. Introduction

As an alternative to Portland cement, geopolymers are produced by activating aluminosilicate-rich sources with alkaline solutions, forming a solid matrix built on interconnected Si–O–Al and Si–O–Si bonds [1]. These binders offer promising mechanical performance and enhanced durability, making them viable alternatives to ordinary Portland cement (OPC) in various civil engineering applications. Unlike OPC, geopolymer production does not involve calcination, thereby significantly reducing CO_2_ emissions and contributing to more sustainable construction practices [2,3,4,5].

Increasing interest in reducing the environmental impact of construction materials has led to intensified research on alkali-activated binders formulated from industrial by-products, including fly ash, metakaolin, and ground granulated blast furnace slag (GBFS) [6,7,8]. Among these, GBFS is particularly attractive for its elevated calcium and silica content, which contributes to early strength development and a denser reaction product [9,10]. Recent work has further highlighted the potential of optimized slag-based systems to achieve both mechanical efficiency and sustainability in construction materials [11]. Beyond structural applications, their chemical stability and capacity to immobilize hazardous species have also led to research on their use in radioactive waste containment and long-term storage [12].

Adjustments in curing temperature have been shown to alter the rate of geopolymer reactions, influence setting behavior, and determine how the material performs over time. While elevated temperatures accelerate polycondensation, excessive heat may cause water loss and microcracking [13,14,15]. Recent studies have shown that ambient-cured geopolymer mortars incorporating GBFS and metakaolin can achieve compressive strengths exceeding 50 MPa, while maintaining resistance to freeze–thaw cycles and chemical attack [16]. Moreover, thermal curing has been identified as a key factor influencing the long-term durability of slag-based alkali-activated materials, particularly in terms of chloride ingress and structural stability [17].

Thermal activation has been widely explored in alkali-activated and geopolymer systems as a means of accelerating dissolution, enhancing polycondensation, and improving early-age strength. Several studies have reported that moderate curing temperatures (30–60 °C) promote denser gel formation and improved mechanical performance, whereas higher temperatures (>60 °C) can induce rapid moisture loss and microcracking, compromising long-term durability [9,13,14,15,17]. Alternative strategies, such as the use of chemical admixtures or hybrid curing protocols, have also been investigated to balance early strength gain with dimensional stability [11,18]. For instance, hybrid activation methods combining moderate heat with tailored admixture addition have been shown to yield high-performance, durable binders while reducing energy input [19].

Previous studies have also examined the influence of curing regimes on the microstructure and performance of alkali-activated slag binders. For example, El-Hassan et al. [20] found that blended slag–fly ash systems subjected to specific water-then-air curing sequences exhibited superior density, mechanical strength, and gel morphology compared to continuous curing methods.

Despite these advances, most prior research has focused on blended systems incorporating multiple precursors, leaving a limited understanding of thermal activation effects in single-precursor GBFS geopolymers. This gap is particularly relevant for optimizing curing protocols that enhance performance while maintaining environmental and economic viability. This study systematically assesses controlled thermal activation across a curing temperature range of 20–80 °C applied exclusively to GBFS-based geopolymers. This approach isolates the effect of heat treatment from other precursors or admixture influences. This approach enables the identification of an optimal curing strategy tailored for GBFS binder systems, supported by both mechanical performance metrics and environmental considerations.

Although the literature extensively covers blended geopolymers made from multiple precursors [14,21,22,23,24,25,26,27], fewer studies have isolated the behavior of single-source systems such as those formulated solely with GBFS. Recent experiments report compressive strengths approaching 69 MPa in GBFS-based binders enhanced with chemical admixtures, even under ambient curing conditions [18], underscoring the combined role of mix composition and curing method.

In this context, the present study examines the influence of temperature-controlled curing protocols on both early-age and long-term properties of GBFS geopolymers. Modified thermal cycles were employed to assess their effects on setting time, shrinkage, porosity, and compressive performance. The results aim to optimize thermal activation strategies and support the implementation of GBFS-based binders in sustainable construction contexts. The novelty of this study lies in its systematic assessment of thermal curing regimes (20–80 °C) applied exclusively to GBFS-based geopolymers, isolating the role of temperature without the influence of blended precursors or admixtures. By integrating mechanical performance, dimensional stability, and environmental metrics, this work provides a unique contribution to the optimization of sustainable binder systems.

## 2. Materials and Methods

### 2.1. Precursor Material and Alkali Activator

Ground granulated blast furnace slag (GBFS), sourced from ECOCEM (Aix-en-Provence, France), served as the precursor in this study. Its chemical composition was assessed via X-ray fluorescence (XRF) and is summarized in Table 1, in agreement with supplier data [28]. The slag consisted primarily of CaO and SiO_2_, with secondary contributions of Al_2_O_3_ and MgO, while minor oxides (Fe_2_O_3_, Na_2_O, TiO_2_, MnO, SO_3_) were present in trace levels.

**Figure 1:**
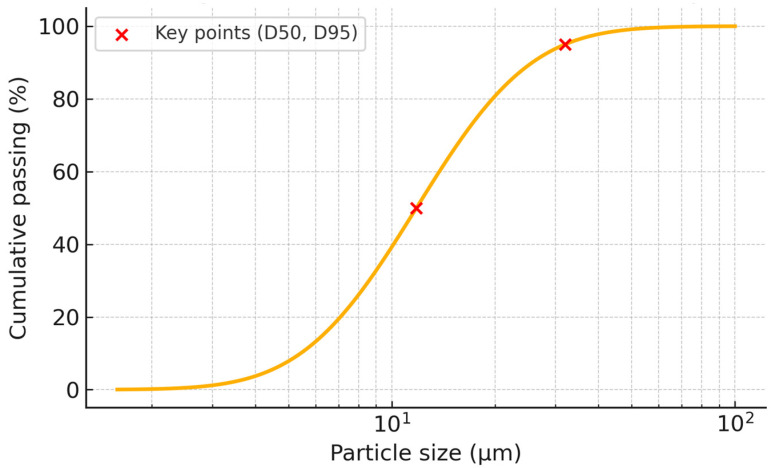
Cumulative particle size distribution of GBFS precursor, based on supplier data.

Physically, the slag exhibited a median particle size (D50) of 11.8 μm, with 95% of the particles below 32 μm. The particle size distribution curve (Figure 1) is based on laser diffraction data provided by ECOCEM for the same production batch. Its Blaine specific surface area was 4450 ± 250 cm^2^/g, and the apparent density was 0.8 ± 0.1 g/cm^3^. The loss on ignition was determined to be 0.7 wt%, indicating minimal residual volatiles. These physical parameters are summarized in Table 2.

Although quantitative XRD was not performed on the specific batch used, GBFS from similar European sources and with comparable chemistry typically contains 91–94 wt% amorphous (glassy) phase and 6–9 wt% crystalline phases, mainly merwinite and akermanite [29,30,31]. According to the supplier’s quality control data [28], the tested slag contained approximately 95% vitreous phase, consistent with typical European GBFS. The high glass content is a key factor in the reactivity of slag in alkali-activated systems, as it facilitates the rapid dissolution of Si and Al species in alkaline media. While variability in glass content and chemistry across sources can influence gel chemistry and mechanical performance, the consistent production process of ECOCEM GBFS, combined with controlled activator composition and curing regime, supports the reproducibility of the results.

The alkaline solution used as activator was composed of 10 molar sodium hydroxide (NaOH) and a commercial sodium silicate (Na_2_SiO_3_) solution with the following composition: 8 wt% Na_2_O, 27 wt% SiO_2_, and 65 wt% H_2_O. These components were blended in a mass ratio of 1:2 (NaOH:Na_2_SiO_3_), resulting in a solution with a molar ratio of Na_2_O to SiO_2_ equal to 0.7 and an overall water content of 66.7 wt%. The activating solution was left to thermal and chemical stabilization under ambient conditions for 24 h prior to mixing.

### 2.2. Composition and Preparation of Geopolymer Paste

For mixture design, a solid-to-liquid (S/L) mass ratio of 2.0 and an activator-to-binder (A/B) ratio of 0.50 were adopted, as these proportions have been shown in previous studies to balance workability, control shrinkage, and promote strength gain in GBFS-based systems [3,4,13,29], mainly by enhancing particle packing and binder densification. The mixture comprised 1458.3 kg/m^3^ of GBFS, 335.3 kg/m^3^ of activator solution, and 208.3 kg/m^3^ of water [32].

The paste contained only GBFS as the precursor, allowing isolation of the slag reaction product (modified C–(A)–S–H) without interference from calcined aluminosilicates, and following prior optimization with the same materials and protocol [29]. The binary alkaline activator (10 M NaOH solution and commercial sodium silicate solution, 8 wt% Na_2_O–27 wt% SiO_2_–65 wt% H_2_O) was prepared at a NaOH:Na_2_SiO_3_ mass ratio of 1:2, providing sufficient alkalinity and soluble silicate for rapid dissolution and gel growth, while avoiding excessively fast setting or efflorescence, as reported for GBFS binders [3,4,13,33].

The GBFS precursor was initially mixed in dry form for 3 min to promote uniform particle distribution. Then, the alkaline activator and additional water were incorporated, followed by another 3 min mixing phase. All procedures were conducted at a controlled temperature of 20 °C. The resulting paste was cast into stainless steel molds (4 cm × 4 cm × 16 cm) and compacted in accordance with ASTM C490 [34].

### 2.3. Curing Regimes

Specimens cured at the ambient baseline temperature (20 °C) were stored in a controlled chamber at 50 ± 5% relative humidity. All samples underwent a 24 h ambient curing stage prior to demolding to ensure sufficient initial setting.

For thermal curing at 40 °C, 60 °C, and 80 °C, a modified theoretical cycle was applied following Leklou et al. [35]: an initial pre-cure at 20 °C for 1 h, temperature ramp-up over 3 h, isothermal hold at the target temperature for 10 h, and gradual cooling to 20 °C over 10 h.

The temperature levels were selected based on their known influence on slag-based geopolymer systems. Literature has reported that the most favorable curing conditions for slag–alkali-activated products are within a curing temperature range of 30 °C to 60 °C and optimal geopolymerization was achieved by curing temperatures ranging from 40 °C to 85 °C within a short time [16,36]. Curing at 40 °C provides moderate acceleration, promoting gel densification while limiting microstructural instability; 60–80 °C were chosen to explore the onset of performance penalties, such as rapid water loss and microcracking, as reported for alkali-activated slag binders [9,14]. After curing, specimens were demolded and stored at 20 °C until testing at 7, 14, 28, and 90 days, enabling assessment of both early-age reaction kinetics and later microstructural stabilization, trends commonly observed in GBFS-based binders [4]. The experimental program is summarized in Table 3. Four curing temperatures (20, 40, 60, and 80 °C) were investigated at four testing ages (7, 14, 28, and 90 days). Each condition was evaluated with three replicate specimens.

### 2.4. Experimental Program

Workability of the fresh paste was evaluated by the flow table method, as outlined in ASTM C1437-15 [37]. Setting times were determined according to ASTM C191-01 [38] and NF EN 196-3 [39], using a manual Vicat apparatus, with three replicate measurements per condition to account for variability.

Shrinkage and dynamic modulus were measured using an extensometer (±1 μm) and impulse excitation technique (Grindosonic^®^, Leuven, Belgium), in accordance with ASTM E1876 and ISO 12680-1 [40,41]. Three prismatic specimens (25 mm × 25 mm × 285 mm) were prepared for each curing temperature, and measurements began 0.5 h after demolding and continued for 90 days. For all tests, curing age (7, 14, 28, and 90 days) was calculated from the moment of mold filling (casting), considered as day 0.

Porosity and bulk density were evaluated at 28 days, in accordance with NF P 18-459 [42], using buoyant mass, saturated-surface-dry mass, and oven-dry mass at 105 °C, with three replicate specimens per curing condition and age. Mechanical strength was evaluated at 7, 14, 28, and 90 days through flexural and compressive tests, performed using a Cyber-Plus Evolution apparatus (Controlab, Saint-Ouen Cedex, France) and following the procedures outlined in NF EN 196-1 [43]. Three prisms (40 mm × 40 mm × 160 mm) were cast for each curing regime and tested under three-point bending. After fracture, both parts from each specimen were used in compressive tests, resulting in six measurements for each condition and age.

Chemical composition of the GBFS was determined using X-ray fluorescence (XRF) spectroscopy (PANalytical Axios mAX, Malvern Panalytical, Almelo, The Netherlands), operating at 4 kW with a Rh anode and SuperQ4 software for elemental quantification. Calibration was performed with certified reference materials, and results were expressed as oxide weight percentages.

Scanning electron microscopy (SEM) images from prior work [32] were used to support the discussion of gel morphology. Analyses were performed using a JEOL JSM-6610LV microscope (JEOL Ltd., Tokyo, Japan) at 15 kV. Samples were gold-coated to minimize charging, and micrographs were taken in secondary electron mode. SEM–EDS was used for local, semi-quantitative oxide estimation of the gel matrix at day 3 (standardless ZAF; area analysis on polished sections). Values were normalized to 100 wt% oxides; light-element quantification (e.g., B) was not considered reliable and is not reported.

Visual inspection of geopolymer specimens was carried out by cutting them at the specified curing ages and photographing the freshly exposed cross-section under consistent lighting conditions. The contrast between reacted and unreacted zones was used as a qualitative indicator of the geopolymerization front.

To evaluate the influence of curing temperature and specimen age on mechanical strength, statistical analyses were performed using two-way and one-way ANOVA, followed by the Tukey–Kramer post hoc test, for pairwise comparisons [44,45]. All analyses were carried out with a significance level of 5%.

All tests were conducted in compliance with international standards to ensure reproducibility and comparability of the results. A summary of the standards applied to each property is presented in Table 4. For the results, values represent averages of three specimens; error bars correspond to ±1 standard deviation. It should be noted that no direct spectroscopic identification of the geopolymeric gel (e.g., XRD, FTIR, NMR) was performed in this study. This represents a limitation, and interpretations are based on bulk XRF, SEM/EDS, and comparisons with literature reports. Future work should address gel identification and phase assemblage to refine these conclusions.

## 3. Results and Discussion

### 3.1. Influence of Thermal Conditions on Initial Geopolymerization and Setting Time

**Figure 2:**
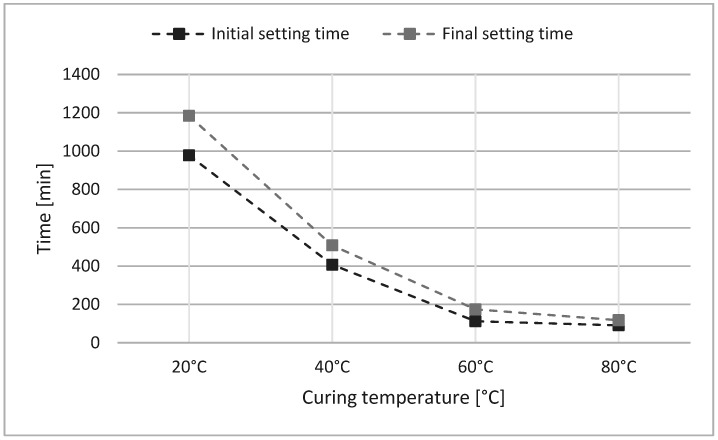
Comparative setting times (initial and final) of geopolymer pastes exposed to distinct curing temperatures.

The fresh geopolymer paste exhibited a slump of 159 mm (±6.62 mm) at ambient conditions. As expected, increasing the curing temperature significantly reduced both initial and final setting times (Figure 2). Elevating the temperature from 20 °C to 40 °C halved the setting time (206 min to 101 min). At 60 °C and 80 °C, the setting times decreased further to approximately 60 min and 30 min, respectively. This behavior is consistent with the accelerated polycondensation reactions at elevated temperatures [46].

**Figure 3:**
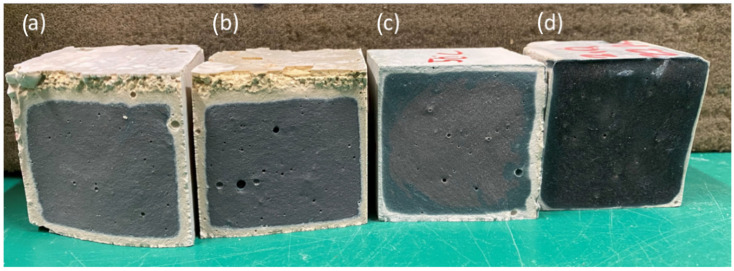
Cross-sectional view of geopolymer specimens one day after mixing, following thermal curing at (**a**) 80 °C, (**b**) 60 °C, (**c**) 40 °C, and ambient curing at (**d**) 20 °C. Darker regions represent more advanced geopolymerization, while lighter areas indicate unreacted or partially reacted zones.

**Figure 4:**
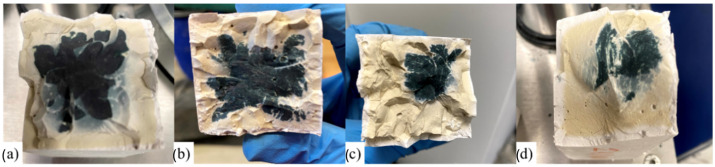
Visual progression of geopolymerization in GBFS-based specimens cured at 40 °C: (**a**) day 2; (**b**) day 3; (**c**) day 4; (**d**) day 5. Darker areas correspond to unreacted or partially reacted regions, while lighter areas indicate advanced geopolymerization.

Visual inspection of specimens after one day of curing at different temperatures revealed progressive geopolymerization, more advanced at higher temperatures. As shown in Figure 3, curing at 80 °C, 60 °C, 40 °C, and 20 °C (Figure 3a–d, respectively) produced distinct differences in reaction extent. For specimens cured at 40 °C, the process was further monitored over several days (Figure 4), showing a progressive increase in the dark-blue reacted zones from day two (Figure 4a) to day five (Figure 4d). These color variations indicate the extent of the reaction front within the specimen, associated with gel formation and densification. This visual tracking method complements mechanical and microstructural analyses by qualitatively revealing the progression of the reaction over time. Surface microcracking was observed in samples cured at 60 °C and 80 °C, likely due to drying shrinkage.

To evaluate microstructural development, XRF analysis (Table 5) of samples cured at 20 °C and 40 °C indicated the presence of aluminum in the latter, suggesting early condensation of the geopolymer gel [14,47]. At 3 days, EDS area analyses targeted the binding matrix rather than unreacted slag particles. Under ambient curing (20 °C), aluminum dissolution is slower, and the gel around the analyzed region showed Ca–Si-rich C–(A)–S–H with Al below the EDS detection limit (reported as n.d. in Table 1). At 40 °C, faster dissolution/precipitation increased Al incorporation into the gel network, resulting in measurable Al_2_O_3_ (7.3 wt%). This local, semi-quantitative snapshot of the matrix does not contradict the bulk Al_2_O_3_ content of the precursor measured by XRF (10.5 wt% in Section 2.1); rather, it reflects early-age heterogeneity and temperature-dependent kinetics. However, bulk XRF analysis of the same batch used in this study confirmed an Al_2_O_3_ content, consistent with typical GBFS values reported in the literature. This confirms that aluminum is present in the raw material and participates in the geopolymerization process, even if not detected in specific early-age gel regions by SEM-EDS.

**Figure 5:**
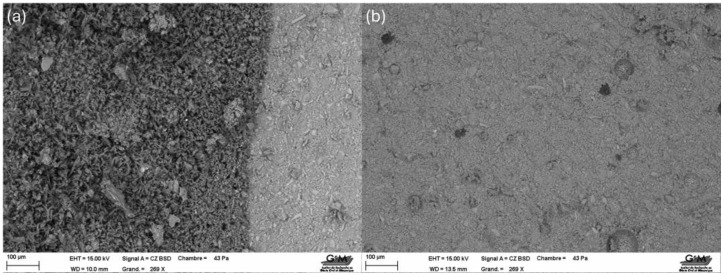
Micrography for samples cured at 20 °C (**a**) and 40 °C (**b**) after one day.

SEM images (Figure 5) further confirmed the advancement of the geopolymer network for the 40 °C-cured samples compared to those at 20 °C, which still appeared to be in the dissolution phase.

Although XRD or FTIR measurements were not performed for the specimens in this specific series, complementary scanning electron microscopy (SEM) coupled with energy-dispersive spectroscopy (EDS) was conducted in a previous study [32] using the same GBFS source, activator composition, and curing protocol. Representative micrographs and spectra revealed the predominance of calcium, silicon, and aluminum, indicating the formation of a modified C–(A)–S–H gel as the primary binding phase. This finding is consistent with the literature on alkali-activated GBFS systems, where calcium-rich environments favor C–(A)–S–H formation over N–A–S–H gels.

Increasing the curing temperature to 40 °C promotes a denser gel network and reduced porosity, whereas further temperature elevation (≥60 °C) may induce partial microstructural instability through rapid water loss, without altering the predominant gel type. These observations align with previous reports on slag-based geopolymers cured under thermal regimes [48,49,50], where temperature chiefly affects gel morphology and packing density rather than phase assemblage.

### 3.2. Mechanical Properties Development

**Figure 6:**
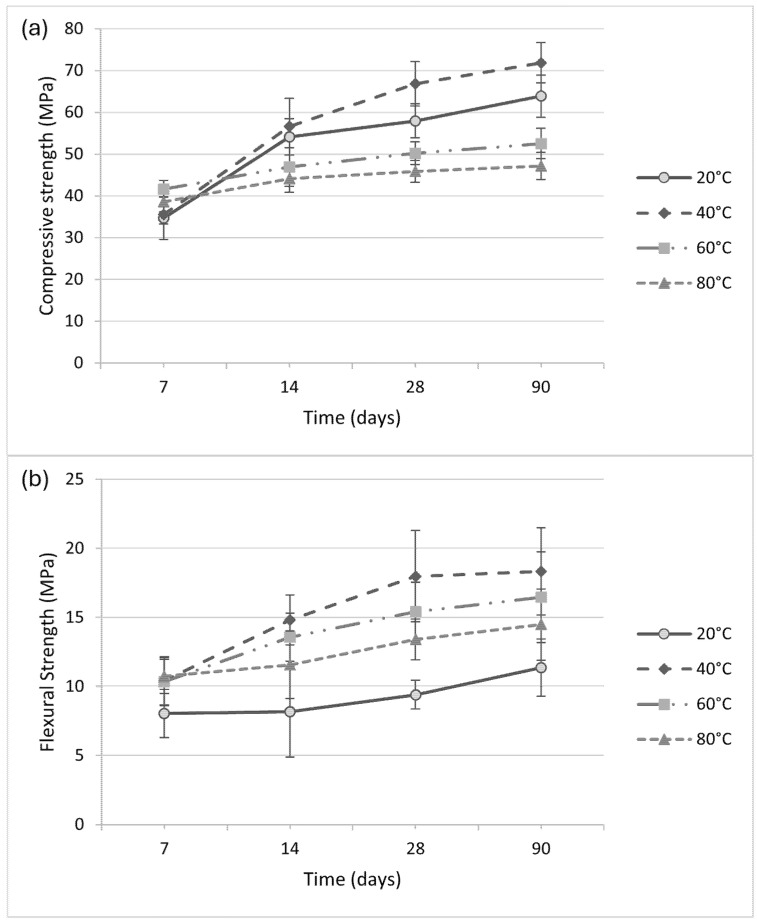
Compressive (**a**) and flexural (**b**) strength development of GBFS-based geopolymers at different curing temperatures.

Figure 6 illustrates compressive and flexural strength evolution at various curing temperatures. At early ages (7 days), samples cured at 60 °C and 80 °C exhibited higher compressive strength (41.66 MPa and 38.53 MPa, respectively) compared to those cured at 20 °C and 40 °C (34.68 MPa and 35.50 MPa). However, from 14 days onward, specimens cured at 40 °C outperformed the others, reaching 71.86 MPa at 90 days. At 40 °C, the curing environment seems to promote better polymeric network formation and minimize stress concentration within the material, which may explain the superior mechanical behavior observed [14,51]. In contrast, excessive curing temperatures may lead to premature gel contraction and reduced long-term strength due to inhibited dissolution [13,15]. Flexural strength showed a similar trend, with all thermally cured samples achieving comparable results for 7 days (~10.5 MPa). However, those cured at 40 °C stabilized at higher values (~18 MPa) from 28 days onward.

In the case of the early age geopolymerization reaction, by elevating the curing temperature, the dissolution of precursors from amorphous phases can be increased, and the hard structure formation can be accelerated [13,40]. In the early stages of geopolymerization, precursor particles dissolve and react to initiate gel formation, gradually reducing pore volume as the network develops. This stage involves the gradual formation of interconnected chains and branches that progressively occupy the pore network, with the process influenced by the presence of water introduced through the alkaline activating solution [52]. The elevated temperature of 40 °C resulted in elevated compressive strength results due to sufficient geopolymer gel bonded during the early age geopolymerization [14].

**Figure 7:**
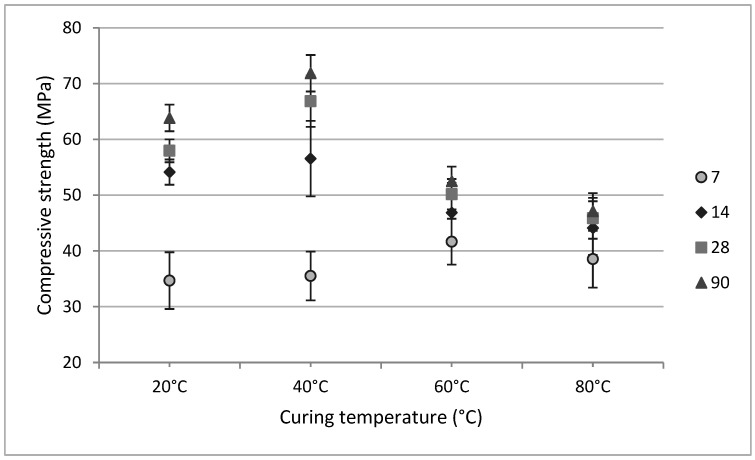
Variation in compressive strength of slag-based geopolymers in different curing temperature conditions.

Increasing the curing temperature from 60 °C to 80 °C resulted in a measurable decline in compressive strength, likely due to premature drying or structural destabilization (Figure 7). From Figure 2, for a curing temperature of 80 °C, at the start of polycondensation, viscosity increased rapidly. Provis and Deventer [15] stated that accelerating the geopolymerization reaction led to undissolved precursor particles covered in geopolymer gel, limiting the dissolution, and hardening. Similar behavior was reported by Khale and Chaudhary [13], who noted a reduction in compressive strength under elevated temperature conditions. The authors stated that curing at higher temperatures or prolonged curing at elevated temperatures resulted in contraction of the gel, breaking the granular structure of the geopolymer mixture, and causing dehydration and excessive shrinkage.

### 3.3. Statistical Analysis of Strength Results

As shown in Table 6, compressive strength was significantly affected by both curing temperature and age (F = 352.46, *p* < 0.01), including a significant interaction between the two variables (F = 32.78, *p* < 0.01). The combination of four curing temperatures (20 °C, 40 °C, 60 °C, and 80 °C) and four testing ages (7, 14, 28, and 90 days) resulted in 16 distinct groups. As summarized in Table 7, a one-way ANOVA was applied to compare all curing groups resulting in an observed F-value of 131.75, which significantly exceeded the critical F-value of 2.35. This outcome supports the rejection of the null hypothesis [44].

The Tukey–Kramer post hoc analysis (Table 8) revealed statistically relevant differences between group means, with specimens cured at 40 °C consistently outperforming those subjected to other temperature conditions. This trend was observed across all tested ages, reinforcing the superior mechanical response associated with 40 °C curing.

### 3.4. Shrinkage and Porosity

**Figure 8:**
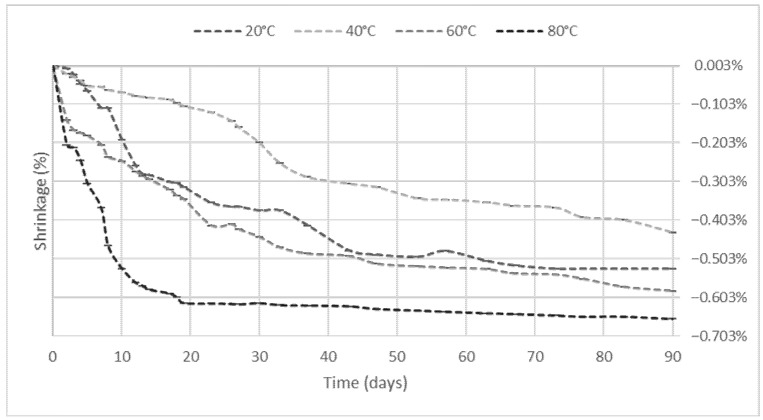
Thermal curing-induced shrinkage in GBFS geopolymer samples over time.

Shrinkage measurements indicated that higher curing temperatures led to rapid water loss and more pronounced shrinkage, especially at 60 °C and 80 °C (Figure 8). This behavior correlates with reduced long-term strength due to microcracking and structural contraction [21,22,53]. In contrast, curing at 40 °C resulted in reduced shrinkage and enhanced strength development.

**Figure 9:**
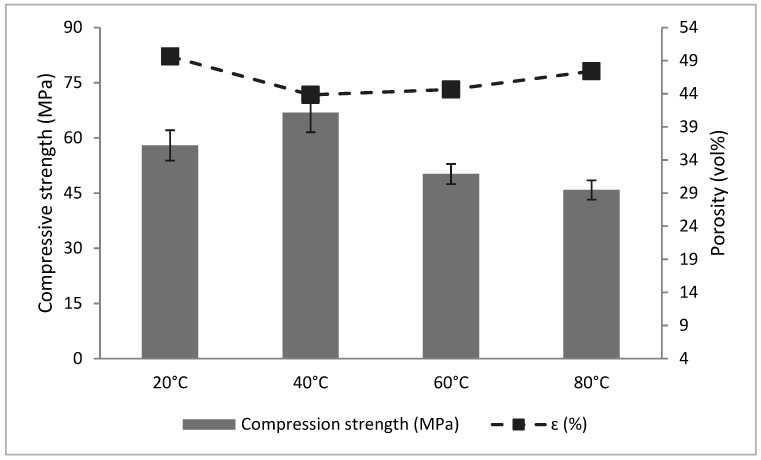
Relationship between porosity levels and compressive strength of thermally cured GBFS geopolymers (28 days).

Porosity measurements at 28 days showed an inverse relationship with compressive strength (Figure 9): specimens cured at 40 °C exhibited the lowest porosity (43.84%) and highest strength (66.85 MPa). Other temperatures yielded higher porosities (44.64% to 49.64%) and correspondingly lower strengths, in agreement with literature findings [54,55].

**Figure 10:**
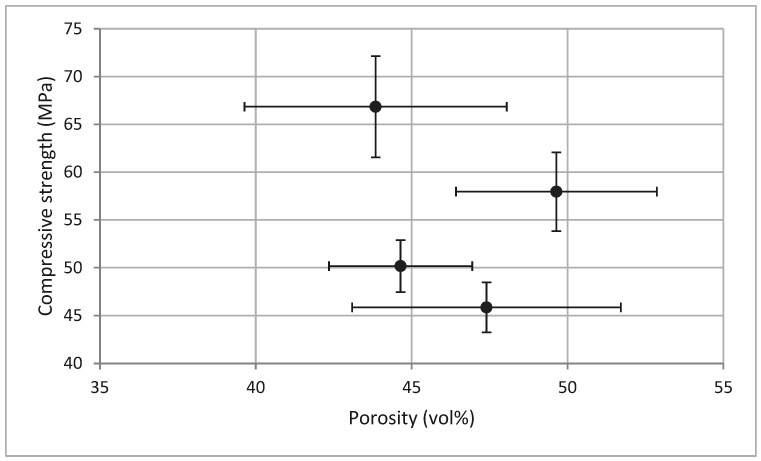
Inverse correlation between compressive strength and porosity in GBFS geopolymers cured at different temperatures (28 days).

Compressive strength increased when the porosity decreased, due to smaller pore sizes. Results for compressive strength vs. porosity at 28 days are presented in Figure 10. As reported by Farhana et al. [54], a reduction in porosity is linked not only to strength improvement but also to increased durability. This behavior was confirmed in the present study, where compressive strength varied inversely with pore volume.

The experimental results show that curing temperature exerts a strong influence on the geopolymerization of GBFS-based binders, affecting both early-age reactivity and long-term performance. The accelerated setting and strength gain observed at 40 °C can be attributed to the enhanced dissolution of the glassy slag phase and increased mobility of dissolved silicate and aluminate species, which promote rapid polycondensation and the formation of a denser C–(A)–S–H-type gel. This effect aligns with prior findings that moderate thermal regimes enhance gel packing density while avoiding microstructural defects linked to excessive water evaporation at higher temperatures.

At temperatures above 60 °C, the reduced strength and increased microcracking are consistent with shrinkage-induced damage caused by rapid moisture loss and possible disruption of gel continuity. These phenomena have been reported in other studies on alkali-activated slag binders, where excessive heat input can compromise the long-term stability of the matrix.

From an application standpoint, the identification of 40 °C as the optimal curing temperature provides a practical, low-energy pathway to producing high-performance GBFS geopolymers. This condition enables compressive strengths above 70 MPa at 28 days, while maintaining low porosity (43.84%) and minimal shrinkage (0.052%). Such performance parameters make these binders suitable for precast elements, repair mortars, and structural applications in which mechanical efficiency, durability, and reduced carbon footprints are equally important. Furthermore, the exclusive use of GBFS as a precursor supports industrial by-product valorization and contributes to circular economy strategies in construction.

### 3.5. Environmental Significance and Energy Considerations

From an environmental standpoint, the thermal curing strategy adopted in this study emphasizes energy efficiency by identifying 40 °C as the optimal activation temperature. The environmental advantage of using GBFS-based geopolymers over ordinary Portland cement (OPC) can be quantified by comparing embodied energy and carbon footprint. Literature data on life cycle assessments report that OPC production requires approximately 3.5–4.6 MJ kg^−1^ of binder, with associated CO_2_ emissions of 0.80–0.95 t CO_2_ eq t^−1^ of cement produced [56,57,58,59]. In contrast, alkali-activated slag binders with thermal curing at moderate temperatures (≤40 °C) show embodied energy values in the range of 1.5–2.5 MJ kg^−1^, and carbon footprints between 0.15 and 0.26 t CO_2_ eq t^−1^ of binder, including precursor grinding, activator production, and curing energy demand [17,59,60,61]. Similar reductions in energy demand and emissions have been reported for sodium carbonate–activated slag systems, with values around 1.8 MJ kg^−1^ and 0.17 t CO_2_ eq t^−1^ [3,6,7].

In this study, the system boundary adopted corresponds to a cradle-to-gate approach, comprising slag grinding, activator production, mixing, curing, and testing preparation, but excluding transportation and end-of-life phases. Regarding allocation rules, the GBFS precursor was considered a by-product of steel production and treated as burden-free up to the grinding stage, in accordance with established practices in life-cycle assessments of alkali-activated binders [16,59].

The additional curing energy was quantified using the following expression:

(1) $$E_{\mathrm{curing}}=\frac{P\times t}{m\times \eta }\;$$

where *P* is the curing oven power (kW), *t* the curing duration (h), *m* the total mass of binder (kg), and *η* the equipment efficiency (assumed at 0.85). Applying this equation yields an additional energy input of approximately 0.14–0.17 MJ·kg^−1^ binder for curing at 40 °C compared to ambient curing [17]. Considering the total energy requirement for the binder (precursor processing + activator production + curing), this represents an increase of less than 8% relative to the lowest reported values for slag-based binders. Importantly, this additional demand is negligible when compared to the avoided emissions from clinker production, translating into a potential CO_2_ savings of approximately 0.65–0.78 t CO_2_ eq t^−1^ of binder.

Furthermore, the exclusive use of GBFS as a precursor contributes to industrial waste valorization and eliminates the demand for energy-intensive calcined additives such as metakaolin, which typically add 1.0–1.5 MJ kg^−1^ to the binder’s embodied energy [17]. This aligns with sustainable construction objectives, as the applied thermal curing strategy not only enhances mechanical performance and durability but also remains consistent with low-carbon pathways for the building sector.

The results obtained in this study align with previous reports on alkali-activated slag binders, where moderate curing regimes (30–50 °C) have been shown to enhance gel densification and mechanical strength [9,14]. In contrast, curing at higher temperatures accelerates drying shrinkage and induces microcracking, compromising long-term performance [13,22]. The identification of 40 °C as an optimal curing temperature is significant. It enables compressive strengths above 70 MPa with low porosity and shrinkage, making it directly applicable in precast construction. Compared to blended systems that combine slag with fly ash or metakaolin, the exclusive use of GBFS simplifies the reaction chemistry and facilitates industrial deployment, while still delivering comparable or superior performance [4,5]. By combining mechanical results with energy and CO_2_ footprint calculations (expressed in kg CO_2_ eq·kg^−1^ binder), this study highlights that GBFS geopolymers can provide both technical and environmental benefits, supporting their adoption as low-carbon alternatives to OPC.

## 4. Conclusions

This study investigated the influence of controlled thermal activation (20 °C, 40 °C, 60 °C, and 80 °C) on the fresh and hardened properties of geopolymers synthesized solely from GBFS. Modified theoretical thermal cycles were applied at each temperature, and their effects on setting time, compressive strength, shrinkage, and porosity were assessed. The geopolymerization of GBFS is considered a silicate and calcium system, and the process occurs with air contact. Variations in curing temperature had a clear effect on geopolymerization. Higher thermal levels accelerated setting and improved early strength, but curing above 40 °C also promoted water loss and microstructural instability, compromising long-term performance.

Within the temperature range investigated (20–80 °C), 40 °C emerged as the optimal curing condition, offering the best balance between strength development, minimal shrinkage, and reduced pore volume. This temperature favored gel formation without the degradation observed at higher thermal exposures. In contrast, specimens cured at 60 °C and 80 °C showed a decrease in compressive strength, with rapid setting time suggesting limitations in the dissolution of amorphous phases that affect hardened geopolymer performance.

The results showed that increasing the curing temperature from 20 °C to 40 °C reduced the initial and final setting times by approximately 35% and 40%, respectively, accelerating early reaction kinetics without causing excessive water loss. The 40 °C curing regime also produced the highest mechanical strengths, with compressive strength values reaching 71.9 MPa and flexural strength of 9.8 MPa at 28 days, remaining stable up to 90 days. In addition, this condition yielded the lowest shrinkage (0.052% after 90 days) and reduced open porosity to about 43.8%, supporting improved durability. Statistical analyses confirmed that both curing temperature and age significantly affected mechanical performance, with 40 °C consistently outperforming the other conditions. From an environmental standpoint, curing at 40 °C adds only about 0.14–0.17 MJ kg^−1^ of additional energy demand compared to ambient curing, while avoiding the high CO_2_ emissions associated with clinker production, reinforcing its potential as a low-carbon alternative for structural binders.

These findings contribute to a better understanding of how thermal curing conditions affect the performance of GBFS-based geopolymers, offering technical support for the development and large-scale application of alternative low-carbon binders. Overall, these results demonstrate that moderate thermal activation at 40 °C optimizes mechanical performance, minimizes shrinkage and porosity, and offers a sustainable route for producing high-performance GBFS geopolymers. This curing regime is directly applicable to industrial-scale precast and repair materials where conditions can be effectively controlled. These results reinforce the importance of optimizing thermal parameters to ensure the structural reliability of GBFS-based geopolymers. These conclusions apply specifically to the tested formulation and curing range. The lack of direct QXRD analysis of the tested batch represents a limitation of this work. However, supplier data (ECOCEM) indicate a vitreous fraction of ~95%, which is consistent with the high reactivity observed. The variability in glassy content may influence reactivity and represent a limitation for reproducibility.

## Figures and Tables

**Table 1:** Chemical composition of GBFS (wt%).

Oxide	CaO	SiO_2_	Al_2_O_3_	MgO	Fe_2_O_3_	Na_2_O	TiO_2_
Content (wt%)	43.2	37.2	10.5	7.0	0.6	0.6	0.5

**Table 2:** Physical properties of GBFS.

Property	Value
Median particle size (D50)	11.8 μm
95% passing size	32 μm
Blaine specific surface area	4450 ± 250 cm^2^/g
Apparent density	0.8 ± 0.1 g/cm^3^
Loss on ignition (LOI)	0.7 wt%

**Table 3:** Summary of the experimental program.

Factor	Levels/Conditions	Properties Evaluated	Testing Ages (Days)
Curing temperature (°C)	20 (ambient), 40, 60, 80	Setting time, shrinkage, porosity, flexural, and compressive strength	7, 14, 28, 90
Replicates	3 prisms per condition		
Specimen dimensions	40 × mm 40 mm × 160 mm (strength tests) 25 mm × 25 mm × 285 mm (shrinkage tests)		

**Table 4:** Properties evaluated in GBFS-based geopolymers and standards applied.

Property/Test	Standard(s) Applied
Flow/workability	ASTM C1437-15
Setting time	ASTM C191-01; NF EN 196-3
Shrinkage	ASTM C490/C490M-09
Dynamic modulus of elasticity	ASTM E1876-15; ISO 12680-1
Porosity and bulk density	NF P 18-459
Flexural and compressive strength	NF EN 196-1

**Table 5:** Oxide composition (wt%) of the geopolymer gel matrix at day 3 obtained by SEM–EDS area analysis.

Temperature	Na_2_O	Al_2_O_3_	SiO_2_	CaO	MnO
20 °C (Sample 1)	6.6	n.d.	24.5	35.7	0.22
20 °C (Sample 2)	6.6	11.9	29.4	33.3	0.32
40 °C	6.6	7.3	23.7	29.2	0.16

**Table 6:** Two-way ANOVA results—compressive strength.

ANOVA	df	SS	MS	F-Value	*p*-Value
Compressive strength	3	10,048.08	3349.36	207.94	0
Curing temperature	3	17,031.16	5677.05	352.46	0
Compressive strength × Curing temperature	9	4752.16	528.02	32.78	0
Error	80	1288.57	16.11	-	-
Total	95	33,119.96	-	-	-

**Table 7:** One-way ANOVA results—compressive strength.

Source of Variation	df	SS	MS	F	Fcrit.
Between groups	15	31,831.4	2122.09	131.75	2.35
Within groups	80	1288.6	16.11	-	-
Total	95	33,120.0	-	-	-

**Table 8:** Tukey–Kramer post hoc comparisons of compressive strength.

Parameter of Analysis	Difference Module
/(7 days and 20 °C–14 days and 40 °C)/	45.81
/(7 days and 20 °C–28 days and 40 °C)/	62.27
/(7 days and 20 °C–90 days and 40 °C)/	70.30
/(7 days and 40 °C–28 days and 40 °C)/	50.15
/(7 days and 40 °C–90 days and 40 °C)/	58.17
/(7 days and 60 °C–90 days and 40 °C)/	48.32
/(7 days and 80 °C–28 days and 40 °C)/	45.31
/(7 days and 80 °C–90 days and 40 °C)/	53.33
/(14 days and 20 °C–90 days and 40 °C)/	60.84
/(28 days and 20 °C–90 days and 40 °C)/	57.02
/(28 days and 40 °C–90 days and 20 °C)/	43.10
/(28 days and 80 °C–90 days and 40 °C)/	41.60
/(90 days and 20 °C–90 days and 40 °C)/	51.13

## Data Availability

The original contributions presented in this study are included in the article. Further inquiries can be directed to the corresponding author.
